# Global research trends and focus on immunotherapy for endometrial cancer: a comprehensive bibliometric insight and visualization analysis (2012-2024)

**DOI:** 10.3389/fimmu.2025.1571800

**Published:** 2025-04-08

**Authors:** Yachen Xu, Tao Wang, Xiaojing Liang, Jie Yang, Yuxiang Zhang, Shan Bao

**Affiliations:** ^1^ Department of Gynecology and Obstetrics, Hainan Affiliated Hospital of Hainan Medical University, Hainan General Hospital, Haikou, China; ^2^ Key Laboratory of Reproductive Health Diseases Research and Translation (Hainan Medical University), Ministry of Education, Haikou, China; ^3^ Hainan Provincial Key Laboratory for Human Reproductive Medicine and Genetic Research, The First Affiliated Hospital of Hainan Medical University, Hainan Medical University, Haikou, China; ^4^ Medical Laboratory Center, Hainan Affiliated Hospital of Hainan Medical University, Hainan General Hospital, Haikou, China; ^5^ School of Public Health, Hainan Medical University, Haikou, China

**Keywords:** endometrial cancer, immunotherapy, bibliometric analysis, research hotspots, emerging topics

## Abstract

**Background:**

This study conducted a novel systematic bibliometric and visualization analysis of global literature on immunotherapy for endometrial cancer (EC) to explore dynamic trends, research hotspots, and emerging topics, providing valuable references for future research.

**Methods:**

Articles and reviews on EC immunotherapy published between 2012 and August 2024 were retrieved from the Web of Science Core Collection (WoSCC). Bibliometric tools, CiteSpace and VOSviewer, were used to analyze clustering patterns and research dynamics.

**Results:**

A total of 861 articles were contributed by 5,331 authors from 1,392 institutions across 58 countries or regions, involving 1,823 keywords. China demonstrated outstanding performance in this field, contributing over 40% of the total publications and ranking first in publication volume. However, the total citation counts for publications from China lags that of the United States, highlighting the latter’s leading position and areas for further improvement in China’s research efforts. The University of Texas Medical Anderson Cancer Center and Nanjing Medical University were the two institutions with the highest number of publications. In terms of authorship, research teams led by Bosse, Tjalling, and Creutzberg, Carien L made significant contributions to advancing the field. Among individual publications, the work by Talhouk et al. achieved the highest average annual citation count of 70.88, demonstrating its profound impact. In terms of journals, *Gynecologic Oncology* emerged as a pivotal academic platform, publishing numerous articles and achieving the highest co-citation frequency. Additionally, *Frontiers in Oncology*, *Frontiers in Immunology*, and *Frontiers in Genetics* have become some of the most active and rapidly developing journals in recent years. Research hotspots are concentrated on themes such as the “Tumor Immune Microenvironment”, “Immune Checkpoint Inhibitors”, and “Targeted Therapy”. Recent trends and frontier research focus on the combined application of immune checkpoint inhibitors with other therapies, research on the application of nanotechnology in immunotherapy, and the integration of artificial intelligence to enhance precision medicine. Additionally, efforts are increasingly directed toward advancing various immunotherapy strategies from basic research to clinical applications.

**Conclusions:**

This comprehensive analysis reveals rapid advancements and significant potential in EC immunotherapy. Strengthening international collaboration and addressing barriers in the translation of research to clinical practice will drive further progress in this promising field.

## 1 Introduction

Endometrial cancer (EC) is a malignancy originating from the uterine epithelium and is the most common gynecological cancer in developed countries, accounting for about 5.9% of all cancers in women (1, 2). Data show that early-stage cancer accounts for approximately 67% of EC cases, with a 5-year survival rate of 81% (3). Among patients diagnosed with metastatic disease, the survival rate is lower (5-year survival rate of 16%), the risk of recurrence is higher, and recurrences often occur outside the pelvis (4). The 5-year survival rate for patients with pelvic recurrences is 55%, but for those with recurrence outside the pelvis, this rate drops to 17% (5). The prognosis of extra-pelvic recurrent disease depends on factors such as disease distribution, molecular subtypes, age, performance status, previous treatments, and the time since the last treatment (4). Overall, various forms of advanced EC still present significant therapeutic challenges (6). Until recently, the treatment of advanced and recurrent EC has changed little, with chemotherapy remaining the mainstay, leaving a large unmet clinical need (7).

Like many other cancers, the tumor microenvironment plays a critical role in the progression of EC and its response to treatment (8, 9). This includes interactions between the tumor and the stroma, as well as between the tumor and infiltrating immune cells (10). In normal endometrium, immune cells play vital roles in defending against external pathogens, promoting fertilization, and supporting tolerance and maintaining pregnancy (11, 12). Certain subtypes of EC exhibit significant immune cell infiltration, suggesting that immunotherapy could be a potentially effective alternative or adjunctive therapy to surgery (8, 13). Therefore, research into immunotherapy for EC is of paramount clinical and practical significance.

The immune microenvironment of the normal endometrium is complex, multi-layered, and adaptable, with immune cell composition and function changing significantly across different stages of the menstrual cycle. Key immune cells include natural killer (NK) cells, dendritic cells, macrophages, T cells (CD4^+^ and CD8^+^), and B cells (14). NK cells are abundant in the endometrium, playing crucial roles in rejecting foreign matter, combating infections, and regulating immune tolerance during pregnancy. They eliminate infected and tumor cells by secreting cytokines such as interferon-γ, and help modulate immune responses to support placental formation and embryo survival (15, 16). Moreover, NK cells exert cytotoxic effects by releasing Granzyme B and Perforin. Perforin forms pores in target cell membranes, allowing Granzyme B to enter and induce apoptosis, eliminating tumors and infected cells (15). In the endometrium, this aids pathogen defense and immune surveillance, supporting uterine stability, infection prevention, and pregnancy success (16). Dendritic cells primarily function as antigen-presenting cells, initiating adaptive immune responses by activating T cells (17). Macrophages participate in local immune defense and are critical for maintaining immune tolerance during pregnancy, secreting cytokines that regulate the endometrial immune environment to prevent fetal rejection (18). T cells in the endometrium include regulatory T cells (Tregs) and effector T cells. Tregs maintain immune tolerance to prevent excessive immune responses against the fetus, while effector T cells recognize and eliminate pathogens (12). During menstruation, the proportion and activity of immune cells increase to clear damaged tissues and cells, while also controlling infections (8). Overall, the immune microenvironment of the endometrium plays a key role in balancing immune defense and tolerance across different physiological stages.

EC is one of the most common malignant tumors of the female reproductive system. It is generally classified into two major types: Type 1 and Type 2. These two types of EC differ significantly in their pathogenesis, clinical manifestations, prognosis, and treatment responses (19). Type 1 EC is typically associated with prolonged exposure to estrogen. It often arises from estrogen-dependent endometrial hyperplasia and is more common in premenopausal or postmenopausal women. Type 1 EC is usually low-grade and well-differentiated, with tumor cells resembling normal endometrial epithelial cells, resulting in a relatively good prognosis. This type of cancer is predominantly hormone-dependent and responds well to hormone therapy, contributing to a relatively favorable outcome. Hormone therapy is often used in the treatment of early-stage EC (20). Type 2 EC is estrogen-independent and typically occurs in older women. These tumors often develop without a clear foundation of endometrial hyperplasia and are characterized by higher malignancy and poorer prognosis. Type 2 tumors are often high-grade, poorly differentiated, and lack significant hormone dependence, exhibiting stronger invasiveness and metastatic potential (21, 22). Unlike Type 1, Type 2 EC typically shows poor response to hormone therapy, necessitating alternative treatments such as surgery, chemotherapy, or radiotherapy.

The immune microenvironment of EC changes significantly as the disease progresses, reflecting the invasiveness and aggression of the tumor. In early-stage (stage I) EC, the immune microenvironment is generally “milder,” with stronger local immune responses. Tumor-associated immune cells primarily include T cells, dendritic cells, and NK cells, all of which play essential roles in tumor recognition and clearance. In stage I EC, the immune system remains relatively effective at detecting and eliminating tumor cells, and the immune response is largely intact (23, 24). However, as the disease progresses into more advanced stages (stages II, III, and IV), the composition and function of immune cells within the tumor microenvironment undergo significant changes (25). As EC progresses, the tumor microenvironment gradually develops a hypoxic state, leading tumor cells to regulate metabolism by activating hypoxia-inducible factor-1α (HIF-1α) to adapt to adverse conditions. Meanwhile, lactate accumulation and alterations in glucose metabolism (the “Warburg effect”) not only provide a growth advantage for tumor cells but also suppress T cell function and promote the recruitment of immunosuppressive cells (26, 27). Additionally, the number of M2 macrophages, regulatory T cells (Tregs), and myeloid-derived suppressor cells (MDSCs) increases, suppressing anti-tumor immunity through the secretion of cytokines such as TGF-β and IL-10 (28). Moreover, the high expression of PD-L1 on tumor cells further weakens T cell activation, promoting immune evasion (29). Furthermore, tumor cells employ various mechanisms to induce immune tolerance, preventing the immune system from recognizing and effectively eliminating the tumor cells (30). In addition to immune evasion, tumor-associated fibroblasts (CAFs) also play a significant role in the immune microenvironment of EC. CAFs promote tumor growth, invasion, and metastasis by secreting growth factors and cytokines that further suppress immune responses (31, 32). As the disease progresses, the immune system within the tumor microenvironment becomes increasingly ineffective at counteracting the tumor, contributing to resistance to treatment and poor prognosis.

In recent years, immune therapy has gained significant attention in the research and treatment of EC. Immunotherapy is based on the idea of harnessing the body’s immune system to recognize and destroy tumor cells. Among the various immune therapies, immune checkpoint inhibitors, particularly PD-1/PD-L1 inhibitors, have emerged as a promising treatment strategy (33). The PD-1/PD-L1 pathway plays a critical role in immune evasion in many tumors, including EC (34). By blocking this pathway, PD-1/PD-L1 inhibitors can restore T cell function and enhance the immune response against tumors. In EC, especially in cases with high microsatellite instability (MSI-H) or defective mismatch repair (dMMR), immune checkpoint inhibitors have demonstrated promising clinical results (4). Studies have shown that high PD-L1 expression is often associated with MSI-H or dMMR endometrial cancer (4). These subtypes have a higher neoantigen load, making them more easily recognized by the immune system and therefore more responsive to immune checkpoint inhibitors (ICIs) (35).These patients typically have higher mutation loads and stronger immune responses, making them more sensitive to immune checkpoint inhibition. Several clinical trials have shown that PD-1/PD-L1 inhibitors significantly improve outcomes in these patients, leading to higher survival rates. In addition to immune checkpoint inhibitors, tumor vaccines, immune cell therapies (such as CAR-T cell therapy), and other immune-modulatory strategies are also under investigation, aiming to activate or enhance the immune system to better target and eliminate tumors (36, 37). Although CAR-T therapy has been successful in hematologic malignancies, its efficacy in solid tumors such as EC is limited by the suppressive effects of the tumor microenvironment (TME) (38). For example, immunosuppressive factors in the TME (such as TGF-β and IL-10), hypoxia, and tumor-associated fibroblasts (CAFs) collectively reduce the survival and cytotoxicity of CAR-T cells (38). Increasing research is exploring the combination of PD-1/PD-L1 inhibitors with other therapies, such as anti-VEGF agents, tumor vaccines, or metabolic interventions, to overcome the immunosuppressive TME and enhance the effectiveness of immunotherapy (3, 24, 39).

While immunotherapy remains an emerging field in the treatment of EC, ongoing basic research and clinical trials are expected to expand treatment options for patients, especially those with advanced or recurrent diseases who have limited responses to conventional treatments. As research progresses, immunotherapy could become a new breakthrough in the treatment of EC, improving patient prognosis and survival rates. Certainly, although researchers have made relentless efforts at the basic research level and proposed various innovative approaches to push their potential for clinical application, a considerable proportion of current immunotherapy strategies still face a lack of widespread, solid evidence of practical value, particularly with significant shortages in clinical settings. The profound barriers between basic research and clinical application highlight significant deficiencies in current research. Therefore, there is an urgent need for researchers in the molecular immune mechanisms of EC and novel immunotherapy strategies to overcome their own knowledge gaps, explore more avenues for collaboration, and find integration points that bridge geographical and disciplinary divides.

Currently, although some reviews have been conducted on the immune microenvironment and immunotherapy strategies for EC, they primarily focus on specific advancements, leaving other relevant aspects unexplored. Thus, there is a need to use specialized tools for a comprehensive analysis of literature in this field, providing researchers with multidimensional guidance and insights. Bibliometrics is a discipline that uses statistical and mathematical tools to analyze bibliographic data, studying the quantity, quality, structure, trends, and impact of academic publications (40, 41). It helps evaluate the development of academic achievements, journals, authors, institutions, and fields. By analyzing large volumes of literature, bibliometrics reveals trends in specific disciplines, allowing researchers to quickly grasp research hotspots and frontier issues (42, 43). It also helps assess academic influence through citation analysis, supporting academic evaluation and funding applications (29, 44). Additionally, bibliometrics uncovers research collaboration networks, identifies key collaborators, and promotes interdisciplinary cooperation. It assists researchers in selecting high-impact journals and aids publishers in evaluating their rankings and influence. Analyzing keywords and themes helps identify knowledge gaps, offering inspiration and guidance for future research directions (45).

The research progress in immunotherapy for EC has provided new molecular-level insights into changes in its immune microenvironment, while laying the foundation for targeted treatments or adjunctive therapies transitioning from basic research to clinical application. However, the varying immunotherapy responses at different stages of the tumor, especially the complexity of advanced and recurrent EC, pose significant challenges for researchers. Thanks to the rapid development of molecular biotechnology and the continuous discovery of emerging potential therapeutic targets, this field has been energized, particularly after 2012, with a large volume of related literature published in the past decade. Considering this, we conducted a novel bibliometric analysis of the literature on this topic, aiming to provide valuable insights into the current status and future research directions by using CiteSpace and VOSviewer—two widely recognized software tools in bibliometric analysis and network visualization (46–48). Given that our search strategy did not retrieve valid data prior to 2012, and papers published after September 2024 are still dynamic updating, we focused on analyzing literature published between 2012 and August 2024. Our goal is to analyze the global mainstream trends in this field by reviewing existing literature, identifying the countries, regions, institutions, journals, and authors that exhibit the highest productivity and influence, and determine emerging topics that will attract attention in future research within this field.

## 2 Materials and methods

### 2.1 Bibliometric data source

**Figure 1:**
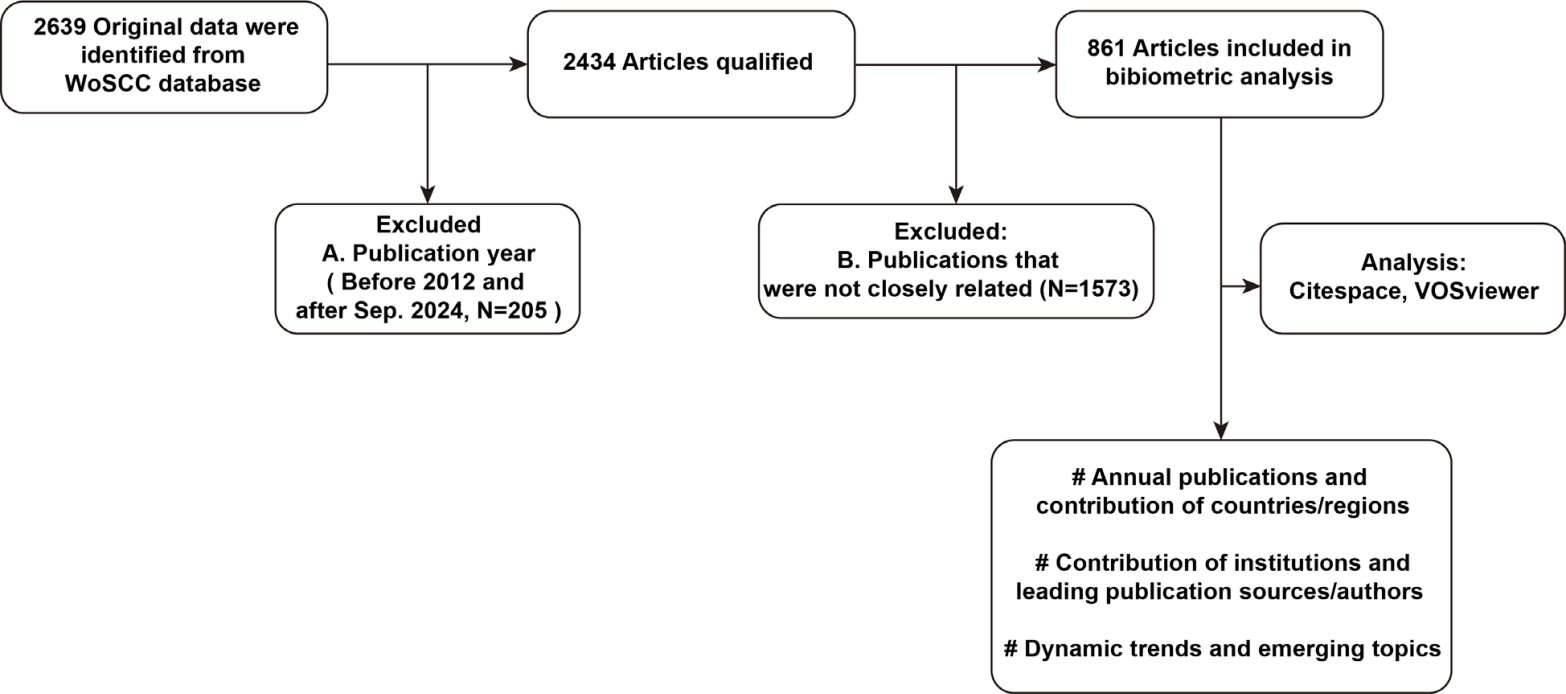
Flowchart depicting the process of literature search and article screening for studies on immunotherapy for EC.

To ensure the accuracy and reliability of the analysis, bibliometric data were obtained from the Web of Science Core Collection (WoSCC) on August 31, 2024, and articles published between 2012 and 2024 were selected for inclusion. In detail, a topic search was conducted with the keywords (“Immun*”) AND (“*therapy”) AND (“Endometrial cancer” OR “Endometrial carcinoma of uterus” OR “Endometrial Carcinoma” OR “Carcinoma of endometrium” OR “Cancer of the Uterine Endometrium” OR “Uterine Cancer”). To enhance the accuracy of the analysis, conference papers and book chapters were excluded, retaining only reviews and research articles. This yielded 2,434 valid results, of which 861 articles remained after removing those unrelated to the topic. The dataset included 1,823 keywords, 5,331 authors, originating from 1,392 institutions across 58 countries or regions. **Figure 1** illustrates the workflow diagram for the literature search and article screening process related to immunotherapy for EC.

### 2.2 Bibliometric analysis

We began by utilizing the “Analyze search results” function in the Web of Science database to systematically organize the annual publication counts, disciplinary distributions, and source journals, aiming to capture the overall trends within the research topic. Subsequently, we exported the basic information of 861 search records for structured management and deeper analysis. Building on this foundation, we employed two analytical tools, VOSviewer 1.6.18 (obtain from https://www.vosviewer.com) and CiteSpace 6.2.R3 (obtain from https://citespace.podia.com), to conduct bibliometric analysis and create knowledge maps. VOSviewer excels at visualizing relationships within scientific domains, offering diverse visualization capabilities, including term relationships, clustering of terms, co-occurrence keyword identification, and dynamic representation of bibliometric and citation networks (49). CiteSpace complements this by uncovering connections between fundamental features of literature, aiding researchers in deciphering the intellectual structure and evolutionary trajectories of the field (50).

In terms of analysis, we explored multiple dimensions, including publication counts, contributing countries, institutions, funding sources, author distributions, subject categories, research areas, and journals. Leveraging established bibliometric methodologies, we further applied visualization techniques to investigate clustering patterns in the field of EC immunotherapy. Specifically, we conducted an in-depth examination of node relationships within each cluster, categorizing them into distinct subcategories and assigning thematic labels to clarify their research focus. Subsequently, we performed a comprehensive cross-analysis of themes and research dimensions, identifying critical research topics and associated concepts within the field. This approach enabled us to delve into the primary research directions and emerging trends, offering a holistic perspective and valuable insights to inform future studies.

## 3 Results

### 3.1 Annual publication trends and country/region contributions

**Figure 2:**
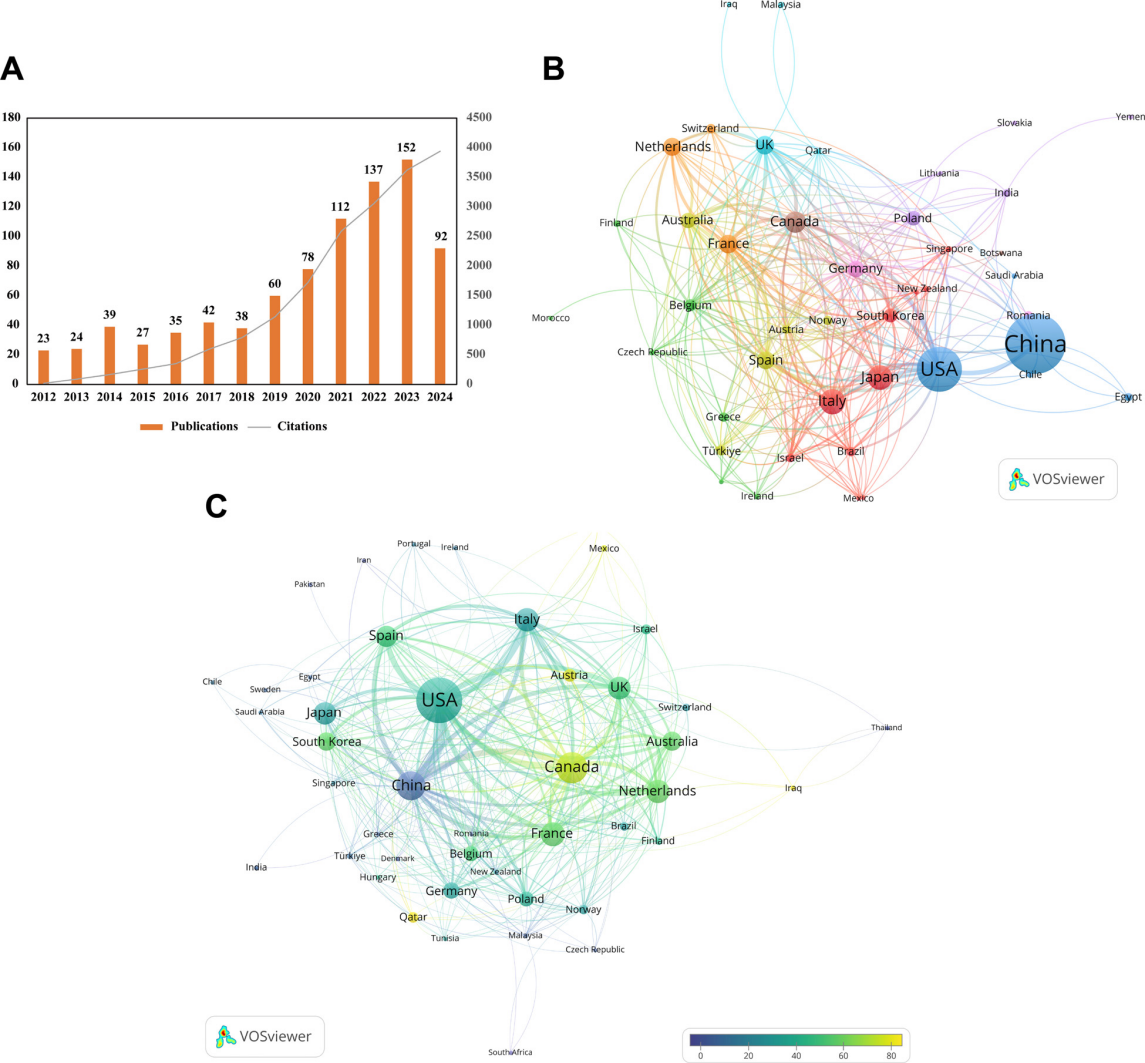
Annual publication trends and country/region contributions. **(A)** Annual publication counts and cumulative citation curves over the past 13 years. **(B)** Country collaboration network map generated by VOSviewer, each country is represented as a node, with links indicating co-authorship affiliations. The size of each node reflects the total number of publications, the more publications, the larger the nodes. The thicker the connections between nodes, the stronger the relationship between the two countries/regions. **(C)** Overlay visualization of country citation analysis created by VOSviewer. The size of the nodes reflects the number of citations—the more citations, the larger the node. The color of the nodes indicates the average citations per paper: the closer to yellow, the higher the average citations; the closer to blue, the lower the average citations. The thicker the connections between nodes, the stronger the relationship between the two countries/regions.

The volume of academic publications reflects the scale and dynamics of development within a research field. As shown in **Figure 2A**, the annual number of publications in this field displayed an upward trend from 2012 to 2023, indicating increasing research interest in this domain. Since 2024 is still ongoing, the available data does not fully reflect the total annual publications for the year. Specifically, in 2023, the annual number of publications and citations in this field reached record highs of 152 articles and 3,623 citations, respectively. Between 2012 and 2023, the total number of citations for all publications in this field amounted to 18,344, suggesting that research on the topic has attracted significant attention over the past 13 years and may see another peak in 2024.

The research contributions from different countries and institutions provide insights into the latest trends in the field. According to **Table 1**, four countries published more than 60 articles: China (353 articles, accounting for 40.99%), the United States (202 articles, 23.46%), Italy (65 articles, 7.55%), and Japan (61 articles, 7.08%). A total of 53 countries/regions have published research on this topic. China leads in publication volume, followed by the United States, reflecting greater research investment and social demand for studies of this filed in these two countries compared to others. However, the total citation count for Chinese articles was 2,952, compared to 7,091 for U.S. articles, with the average citation per article in the U.S. being approximately 26.74 higher than in China. The H-index of Chinese articles was 26, ranking second to the U.S (40)., followed by Italy (23). These figures highlight that U.S. research in this field is more influential, innovative, and impactful. To address this, China should refine its research strategy, enhance international collaboration, and improve the accessibility of its research outcomes on a global scale.

**Table 1:** Top 15 productive countries/regions related to immunotherapy for EC.

Rank	Country	NP	NC	AC	H-index
1	China	353	2952	8.36	26
2	USA	202	7091	35.1	41
3	Italy	65	1870	28.77	24
4	Japan	61	1839	30.15	20
5	Canada	47	3309	70.4	25
6	France	36	1988	55.22	19
7	UK	34	1621	47.68	17
8	Netherlands	34	1921	56.5	22
9	Spain	33	1627	49.3	16
10	Germany	29	933	32.17	11
11	Australia	25	1331	53.24	14
12	Poland	22	823	37.41	7
13	South Korea	21	1162	55.33	12
14	Belgium	16	808	50.5	10
15	Türkiye	11	143	13	6

Ranking: according to the number of total publications. NP, total number of publications; NC, total number of citations; AC, average citations per item.

As globalization progresses, collaboration among countries in the research has become widespread. The national collaboration network in **Figure 2B** indicates that the node size is proportional to publication volume, and the thickness of connections between nodes reflects the strength of collaboration between countries/regions. Saudi Arabia and India, as well as China and the United States, demonstrated the strongest collaborations, with link widths of 49 and 43, respectively, indicating highly active cooperation. Further analysis revealed that countries with total link strengths exceeding 100 include the United States (160), Canada (117), and France (111), with the U.S. significantly surpassing others. This strong collaboration network may partially explain the high influence of the U.S. in this field. However, some countries, such as Iran, Pakistan, and Portugal, have weaker collaborations, as indicated by their total link strength of zero, and do not appear in the figure. Strengthening cooperation with these countries could further advance the research.

Using VOSviewer, the citation overlay visualization of countries (**Figure 2C**) provides additional insights into their contributions to the research. Node size represents the total citation count, while node color reflects the average citation per article. China and the United States occupy central positions, with Canada exhibiting the highest average citation per article at 70.4, far exceeding other countries. This suggests that Canadian research in this field is of exceptionally high quality on average. Although countries such as Iraq, Qatar, Mexico, and Austria have lower publication volumes, their articles are of high average quality, as indicated by the yellowish color of their nodes.

### 3.2 Institutional contributions and leading publication venues

**Figure 3:**
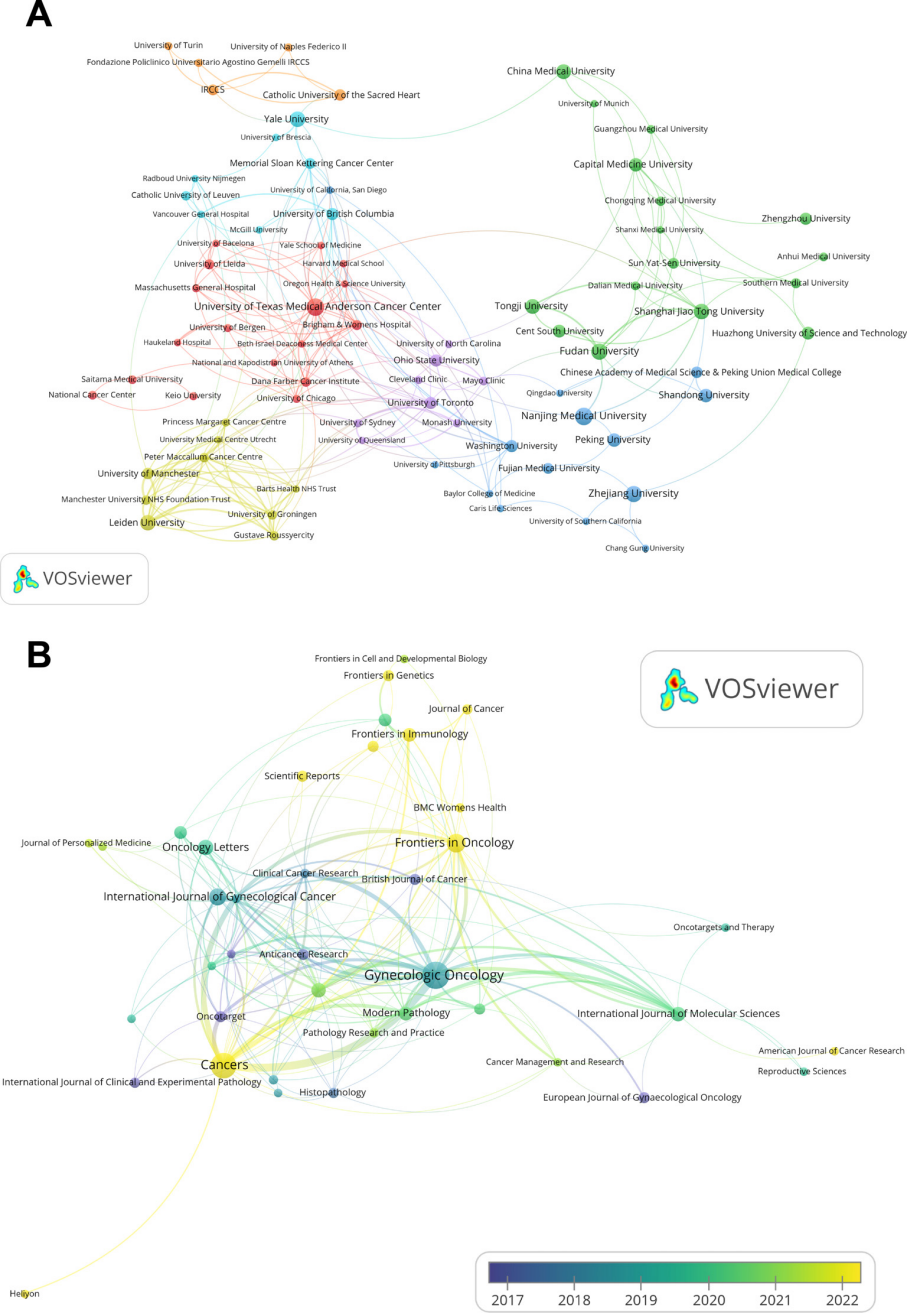
Institutional contributions and leading publication venues. **(A)** Institutional collaboration network map generated by VOSviewer, where node sizes correspond to publication quantities, the thicker the connections between nodes, the stronger the relationship between the two institutions. **(B)** Overlay visualization of journal citation analysis generated by VOSviewer, the color spectrum, ranging from purple to yellow, represents the temporal proximity of publications to either 2017 or 2022, the thicker the connections between nodes, the stronger the relationship between the two journals.

As shown in **Table 2**, the University of Texas Medical Anderson Cancer Center and Nanjing Medical University lead in publication volume, each contributing 26 articles. The institutional collaboration network (**Figure 3A**) reveals that 92 institutions have published at least five papers on this topic. However, international collaboration between institutions from different countries remains limited, with most partnerships occurring among domestic institutions.

**Table 2:** Top 10 most active institutions of publications in immunotherapy for EC field.

Rank	Country	NP	NC	AC	H-index
1	University of Texas Medical Anderson Cancer Center	26	1032	39.69	17
2	Nanjing Medical University	26	338	13.00	13
3	Zhejiang University	22	115	5.23	7
4	Fudan University	21	290	13.81	8
5	Leiden University	19	1023	53.84	15
6	Shanghai Jiao Tong University	19	344	18.11	10
7	Yale University	19	650	34.21	18
8	China Medical University	18	162	9.00	8
9	Tongji University	18	162	9.00	7
10	Capital Medicine University	15	61	4.07	5

Ranking: according to the number of total publications. NP, total number of publications; NC, total number of citations; AC, average citations per item.

In addition, **Table 3** summarizes the top 10 funding agencies actively supporting research in this field. Over half of these funding sources originate from China and the United States, reflecting the significant contribution of these two countries to advancing research in this domain.

**Table 3:** Top 10 funding industries in immunotherapy for EC field.

Rank	Foundation	Country	NP	NC	AC	H-index
1	National Natural Science Foundation of China	China	122	1375	11.27	19
2	National Institutes of Health USA	USA	65	2927	45.03	25
3	United States Department of Health Human Services	USA	65	2927	45.03	25
4	NIH National Cancer Institute	USA	33	1447	43.85	17
5	Ministry of Education Culture Sports Science and Technology Japan	Japan	30	544	18.13	15
6	Japan Society for the Promotion of Science	Japan	28	491	17.54	14
7	Grants in Aid for Scientific Research	Japan	27	484	17.93	14
8	KWF Kankerbestrijding	Netherlands	11	935	85	11
9	Spanish Government	Spain	10	232	23.2	6
10	Natural Science Foundation of Shanghai	China	9	149	16.56	5

Ranking: according to the number of total publications. NP, total number of publications; NC, total number of citations; AC, average citations per item.

The journal citation network (**Figure 3B**), visualized using VOSviewer, includes 39 journals with at least five publications each from 2012 to 2024. Collectively, the top 10 journals published 221 articles on immunotherapy for EC (**Table 4**). *Gynecologic Oncology* ranks first, with 51 publications, 1,317 citations, and a total link strength of 89, leading across all metrics. *Cancers* ranks second with 43 publications, followed by *Frontiers in Oncology* (23) and the *International Journal of Gynecological Cancer* (19).

**Table 4:** Top 10 most productive journals in immunotherapy for EC field.

Rank	Journal	ISSN	Country	IF-2024*	NP	NC	H-index
1	Gynecologic Oncology	0090-8258	USA	4.5	51	1317	21
2	Cancers	2072-6694	Switzerland	4.5	43	530	13
3	Frontiers in Oncology	2234-943X	Switzerland	3.5	24	126	7
4	International Journal of Gynecological Cancer	1048-891X	UK	4.1	20	270	9
5	Oncology Letters	1792-1074	Greece	2.5	16	136	8
6	International Journal of Gynecological Pathology	0277-1691	USA	1.6	15	204	7
7	International Journal of Molecular Sciences	1661-6596	Switzerland	4.9	15	257	8
8	Frontiers in Immunology	1664-3224	Switzerland	5.7	13	135	7
9	Modern Pathology	0893-3952	UK	7.1	13	534	10
10	BMC Cancer	1471-2407	UK	3.4	11	176	6

Ranking: according to the number of total publications. *NP, total number of publications; IF, impact factor; NC, total number of citations.

The overlay visualization of journal citations (**Figure 3B**) further illustrates publication trends. Node size corresponds to the number of publications, with larger nodes representing higher output. Node color reflects the average publication year, where yellow indicates more recent activity and blue indicates earlier contributions. From the visualization, journals such as *Cancers*, *Frontiers in Oncology*, *Frontiers in Immunology*, and *Frontiers in Genetics* are highlighted in yellow, indicating that they are among the most promising and actively developing journals in the field of immunotherapy for EC research.

### 3.3 Authors, co-cited authors, and subject categories

**Figure 4:**
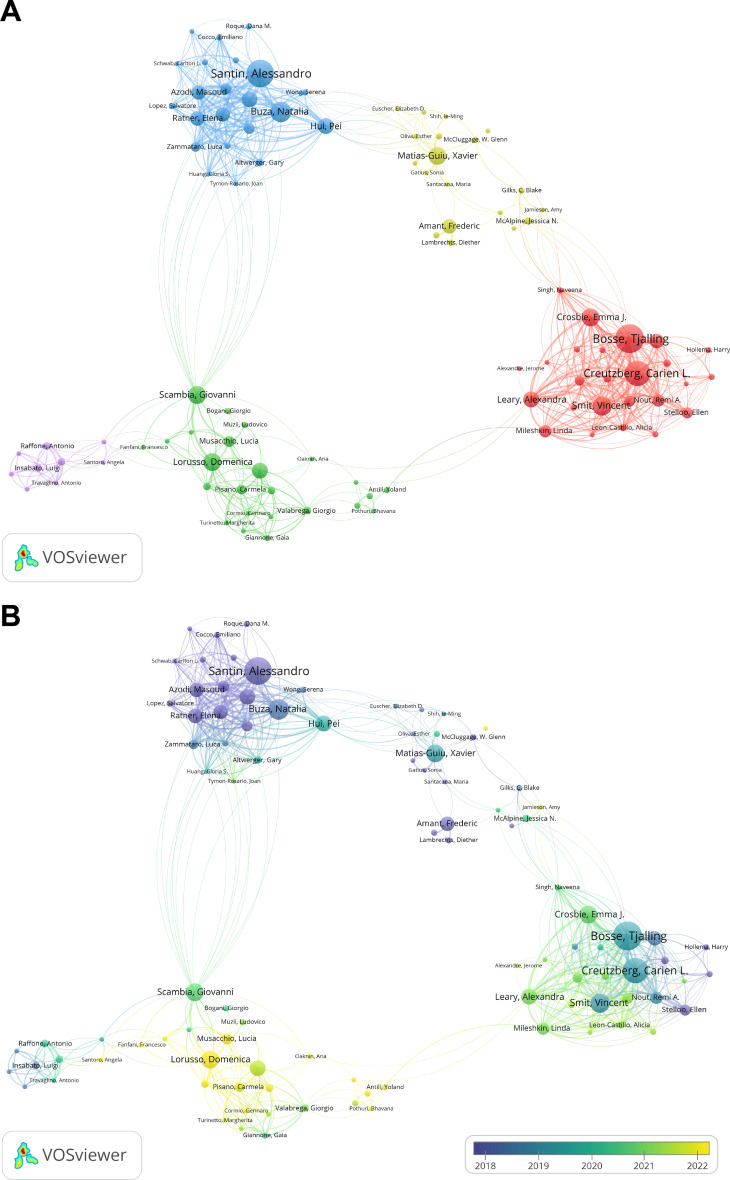
Authors, co-cited authors analysis. **(A)** Author collaboration network map generated by VOSviewer. **(B)** Overlay visualization of author collaborations created by VOSviewer.

According to the author collaboration network map in **Figure 4A** and **Table 5**, scholars with at least three publications are displayed, and they are divided into five major groups: Red group: Represented by Bosse, Tjalling, and Creutzberg, Carien L. Green group: Represented by Lorusso, Domenica and Scambia, Giovanni. Blue group: Represented by Santin, Alessandro. Yellow group: Represented by Matias-Guiu, Xavier. Purple group: Represented by Mollo, Antonio and Raffone, Antonio.

**Table 5:** Top 10 active authors in immunotherapy for EC field.

Rank	Author	Affiliation	NP	NC	AC	H-index
1	Bosse, Tjalling	Leiden University	18	917	50.94	53
2	Santin, Alessandro	Yale University	17	468	27.53	56
3	Creutzberg, Carien L.	Leiden University	16	889	55.56	63
4	Buza, Natalia	Yale University	13	458	35.23	34
5	Smit, Vincent	Leiden University	12	687	57.25	59
6	Crosbie, Emma J.	University of Manchester	11	702	63.82	42
7	Lorusso, Domenica	Humanitas University	11	75	6.82	58
8	Matias-Guiu, Xavier	University of Barcelona	11	405	36.82	66
9	Scambia, Giovanni	Imperial College London	11	117	10.64	82
10	Leary, Alexandra	IRCCS	10	670	67.00	63
11	Hui, Pei	Xidian University	10	220	22.00	44
12	Pignata, Sandro	IRCCS	10	152	15.20	63
13	Schwartz, Peter E.	Yale University	10	263	26.30	55

Ranking: according to the number of total publications. NP, total number of publications; NC, total number of citations; AC, average citations per item.

The red group (red) is centered around the research teams of Bosse, Tjalling and Creutzberg, Carien L. Among them, Bosse, Tjalling from Leiden University has the highest publication count, with 18 papers, an average publication year of 2019, a total citation counts of 909, and a total link strength of 99. This indicates that he started his research in the field early and has had significant influence. Five of the top ten authors in the table belong to this group, indicating that this team is tightly connected, has developed early, and has played a key role in advancing research on this domain.

The author collaboration overlay visualization in **Figure 4B** further reveals the average publication time and research trends of different groups. The third group (blue) is the earliest in terms of publication time, with most research focused around 2017. The second group (yellow) is the most recent, with most publications appearing in 2022.

In the second group, representative scholar Lorusso, Domenica from Humanitas University has published 11 papers, with an average publication year of 2022, a total of 75 citations, and a total link strength of 48. Another representative scholar, Scambia, Giovanni from Imperial College London, also published 11 papers, with an average publication year of 2020, a total of 117 citations, and a total link strength of 43. Although this group has a lower total citation count, the papers’ publication times are closer to the present, resulting in fewer citations. This suggests that the team’s research is still in development and may become a leading force in the research in the future.

**Figure 5:**
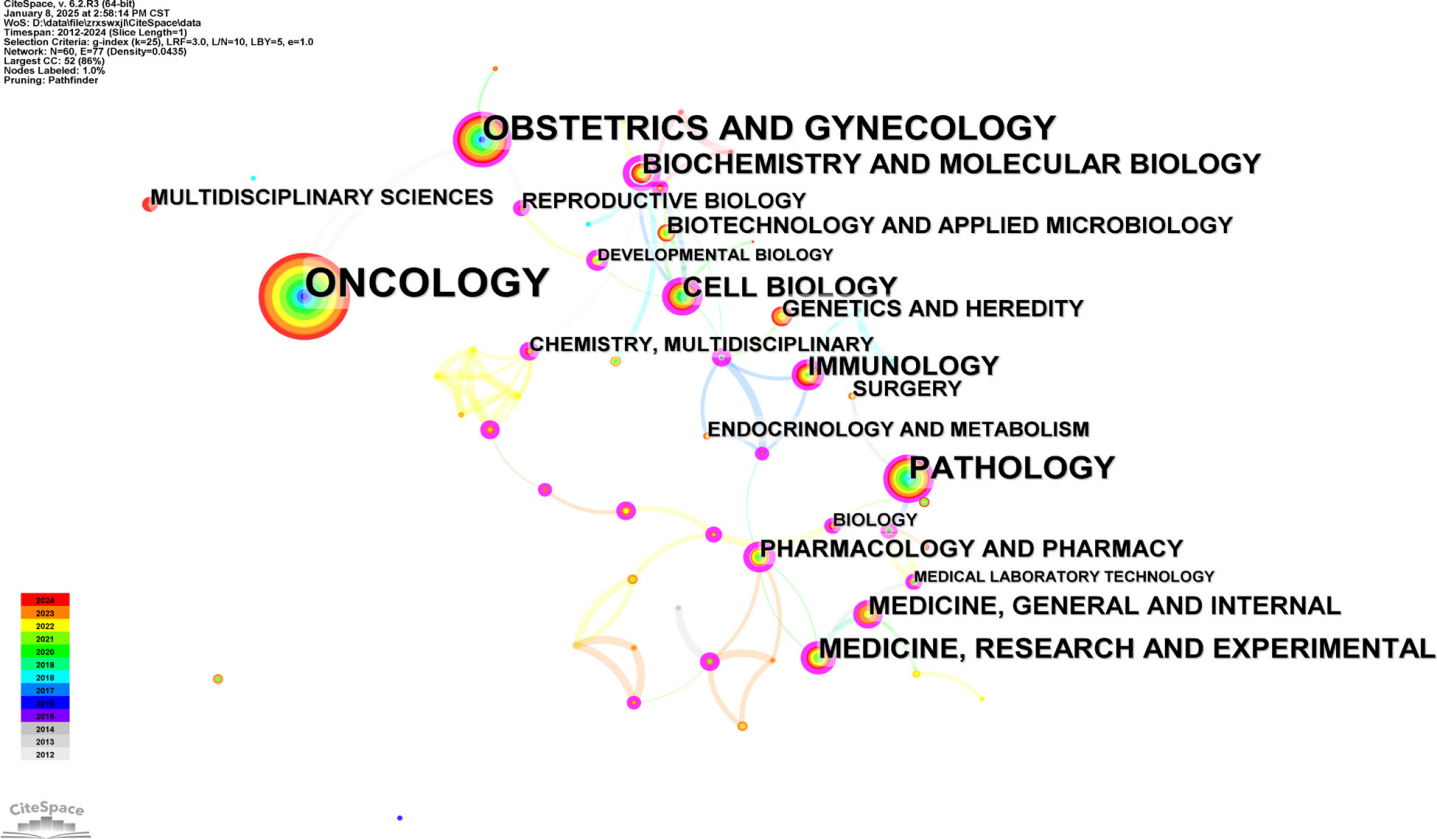
Subject categories analysis. Co-occurrence network analysis of related subject categories created using CiteSpace.

At the same time, **Figure 5** displays a visualization map of co-occurring subject categories, created using Citespace. The top five subject categories, ranked by frequency, are Oncology, Obstetrics and Gynecology, Pathology, Cell Biology, Biochemistry & Molecular Biology, Medicine & Research and Experimental.

### 3.4 Co-cited references, reference bursts, and highly cited publications

**Figure 6:**
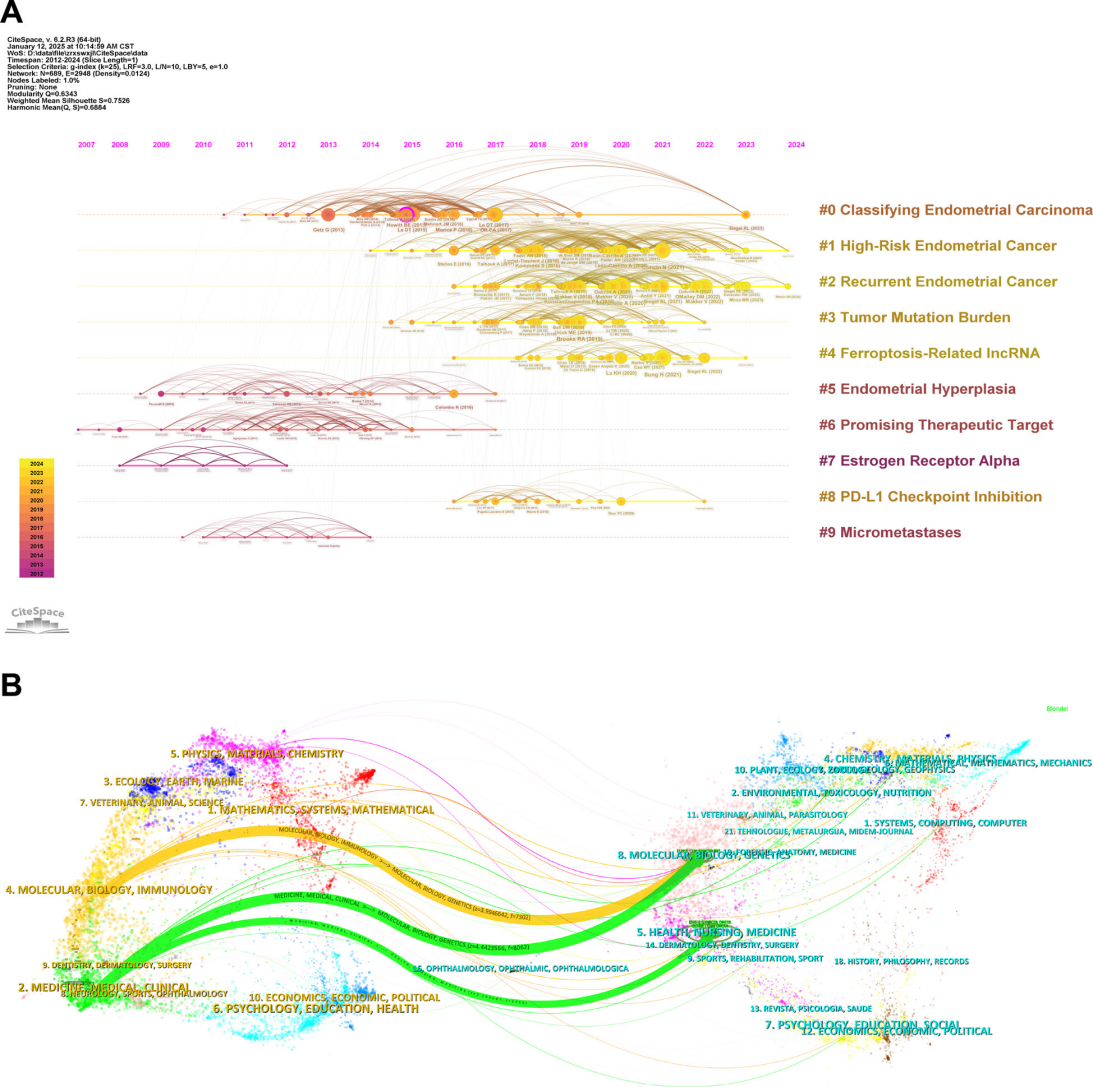
Co-cited references, reference bursts, and highly cited publications. **(A)** Timeline view of reference co-citation analysis generated by CiteSpace. **(B)** Dual map overlay of journals created by CiteSpace, the left side presents the citing journals, while the right side represents the cited journals. Citation relationships are depicted with colored paths, where thicker lines indicate major citation pathways.

In the reference co-citation analysis timeline view generated by CiteSpace (**Figure 6A**), the relationships between two articles co-cited in other publications are depicted. Generally, the analysis of keywords and references is a crucial component of academic research, as it reflects current research hotspots. In this timeline view, the position of a node on the horizontal axis represents the time of its first appearance, while the lines connecting nodes indicate co-citation relationships. The size of a node is proportional to its citation count, with yellow nodes indicating more recent citations closer to 2024 and purple nodes representing earlier citations closer to 2007. This visualization also reveals the evolutionary trajectory of research within the field. References included in the analysis are categorized into nine clusters based on their main research themes. Notably, clusters such as “Endometrial Hyperplasia” (Cluster 5), “Promising Therapeutic Target” (Cluster 6), and “Estrogen Receptor Alpha” (Cluster 7) represent earlier research topics, whereas clusters like “High-Risk Endometrial Cancer” (Cluster 1), “Recurrent Endometrial Cancer” (Cluster 2), “Tumor Mutation Burden” (Cluster 3), and “Ferroptosis-Related lncRNA” (Cluster 4) signify current research focuses.

Furthermore, the dual map overlay of journals (**Figure 6B**) provides additional insights into the relationship between citing and cited journals. The citing journals are displayed on the left, while the cited journals are on the right. Colored paths represent citation links, with thicker paths indicating more frequent citations. This map reflects the disciplinary distribution of academic journals. Two primary citation pathways are observed: the first links journals in the “Molecular, Biology, Genetics” domain to those in the “Molecular, Biology, Immunology” domain, and the second connects journals in the “Molecular, Biology, Genetics” and “Health, Nursing, Medicine” domains to those in the “Medicine, Medical, Clinical” domain. As research in this field progresses, these citation pathways are expected to expand and diversify.

**Figure 7:**
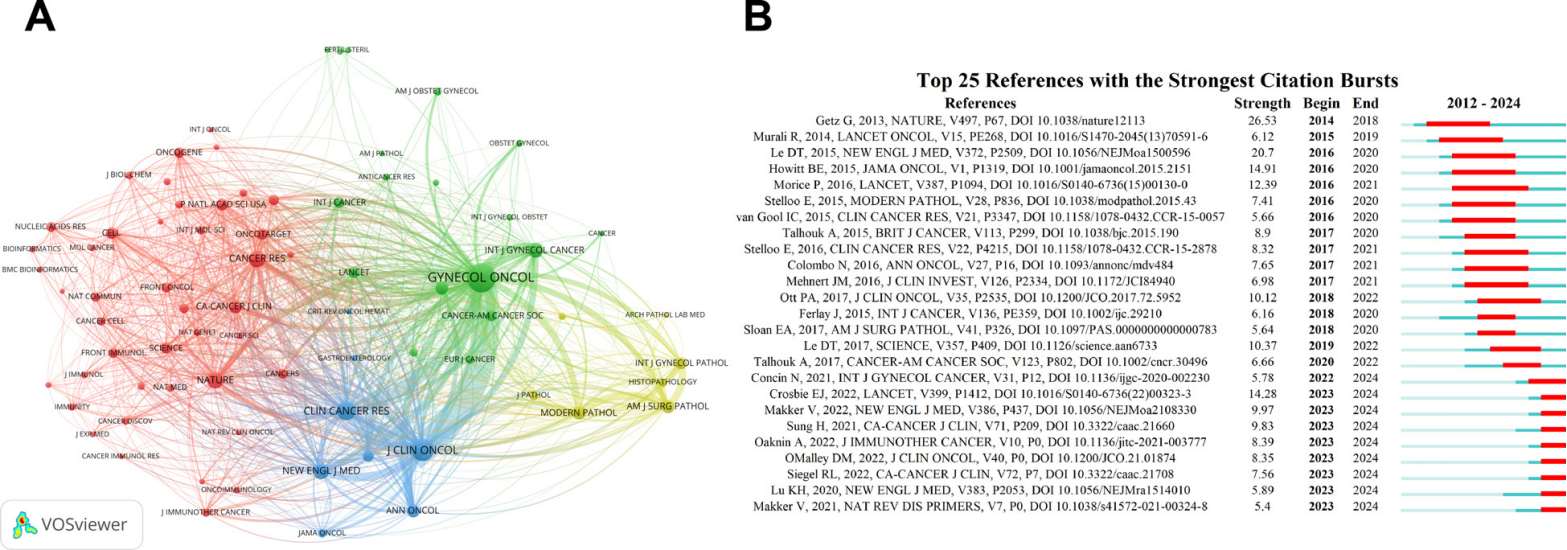
Co-citation network visualization and citation burst analysis. **(A)** Cited sources co-citation network visualization generated by VOSviewer. **(B)** Citation burst analysis identified by CiteSpace, the timeline is indicated by a blue line, and burst periods are represented using red bars. These bars denote the commencement year, conclusion year, and duration of the burst for each reference.

Additionally, CiteSpace was used to analyze citation bursts, which highlight articles experiencing a sudden surge in citations over a specific period, indicating their rapid recognition and dissemination within the field. The network of cited journals is visualized in **Figure 7A**. According to the data, *Gynecology Oncology* exhibits the highest total link strength (90,114) and citation count (2,456). *Journal of Clinical Oncology* follows with a total link strength of 67,589 and 1,600 citations, while *Clinical Cancer Research* has a total link strength of 50,589 and 1,104 citations. These are succeeded by *Nature* (34,810/874) and *Cancer Research* (33,754/874), underscoring their pivotal roles in guiding research and providing foundational references in the field of EC immunotherapy. The top 10 citation bursts within the field, as illustrated in **Figure 7B**, indicate that the first citation burst occurred in 2014, with the most recent burst appearing in 2023. This trend demonstrates that the field remains dynamic and continues to garner significant attention.

Moreover, the higher the citation count of a paper, the greater its academic impact. Analyzing highly cited papers can help identify hotspots within the field. In the context of this study, as shown in the top 10 cited journals (**Table 6**), the 10 most frequently cited papers were selected based on their average annual citation count. The most cited paper, published by Talhouk et al., has an average of 70.88 citations per year. The second most cited paper, authored by Howitt et al., has an average annual citation count of 48. These findings highlight that immunotherapy research in EC is an appealing and trending research topic.

**Table 6:** Top 10 highly cited publications in immunotherapy for EC field.

Rank	Title	NC	AC	Year	RF
1	Confirmation of ProMisE: a simple, genomics-based clinical classifier for endometrial cancer	567	70.88	2017	(51)
2	Association of polymerase e-mutated and microsatellite-instable endometrial cancers with neoantigen load, number of tumor-infiltrating lymphocytes, and expression of PD-1 and PD-L1	480	48.00	2015	(52)
3	Molecular classification of the portec-3 trial for high-risk endometrial cancer: impact on prognosis and benefit from adjuvant therapy	427	85.40	2020	(53)
4	Assessing tumor-infiltrating lymphocytes in solid tumors: a practical review for pathologists and proposal for a standardized method from the international immuno-oncology biomarkers working group: part 2: tils in melanoma, gastrointestinal tract carcinomas, non-small cell lung carcinoma and mesothelioma, endometrial and ovarian carcinomas, squamous cell carcinoma of the head and neck, genitourinary carcinomas, and primary brain tumors	405	50.63	2017	(54)
5	Current recommendations and recent progress in endometrial cancer	392	65.33	2019	(55)
6	Lenvatinib plus pembrolizumab in patients with advanced endometrial cancer: an interim analysis of a multicentre, open-label, single-arm, phase 2 trial	359	59.83	2019	(56)
7	Safety and antitumor activity of pembrolizumab in advanced programmed death ligand 1-positive endometrial cancer: results from the keynote-028 study	352	44.00	2017	(57)
8	Proteogenomic characterization of endometrial carcinoma	271	54.20	2020	(58)
9	Enhanced expression of PD-L1 in cervical intraepithelial neoplasia and cervical cancers	209	20.90	2015	(59)
10	Pembrolizumab plus chemotherapy in advanced endometrial cancer	190	95.00	2023	(60)

Ranking: according to the number of total publications. NC, total number of citations; AC, average citations per item; RF, references.

### 3.5 Keyword co-occurrence analysis and emergent keywords display

Keywords are a crucial component of academic papers, encapsulating their essence, and keyword analysis serves as an essential indicator for identifying research hotspots. Conducting bibliometric analyses of literature can help elucidate the evolution of this field and forecast future research trends and focal areas. In this study, two bibliometric tools were used for a comprehensive analysis.

**Figure 8:**
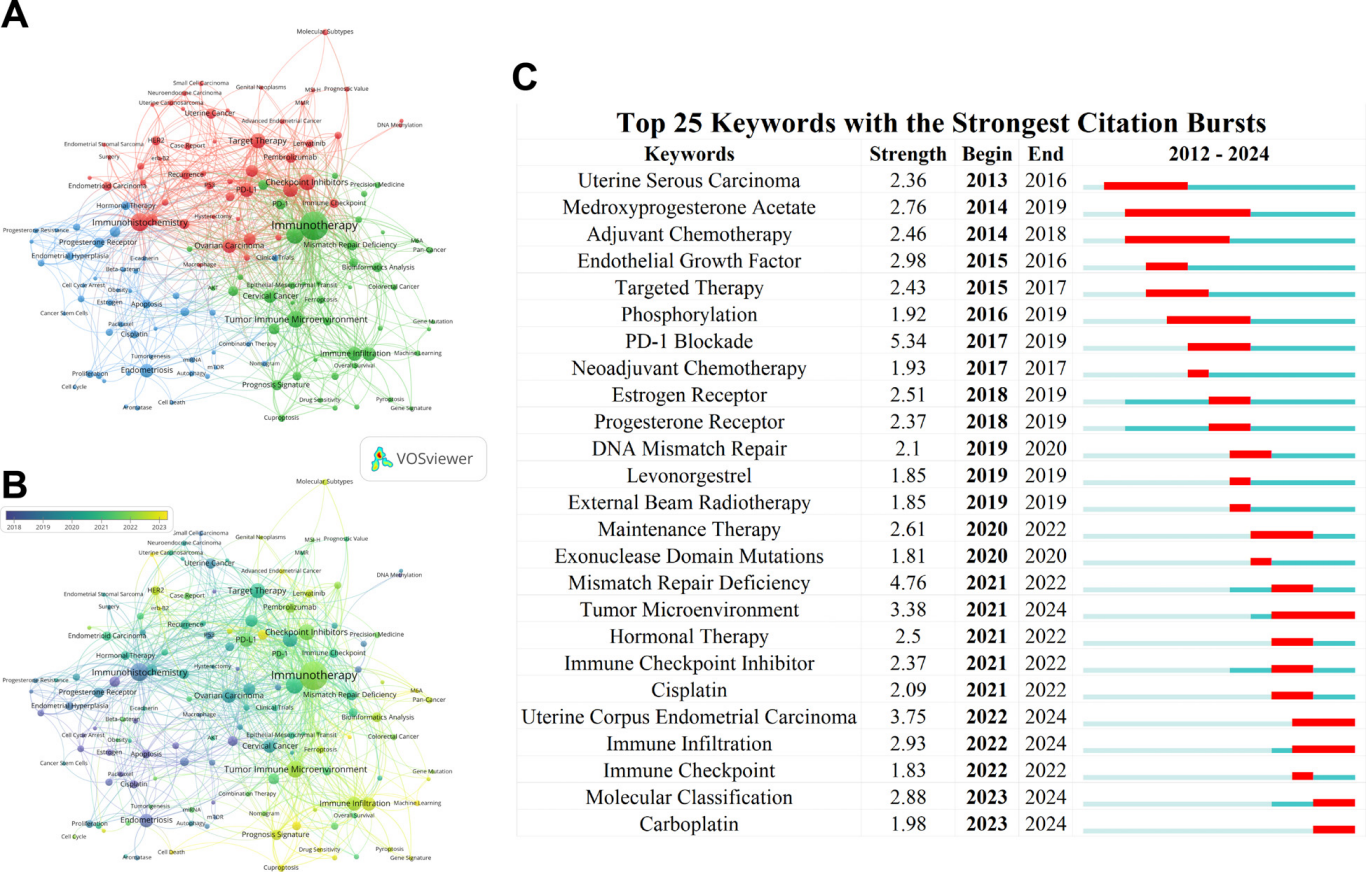
Keyword co-occurrence analysis and emergent keywords display. **(A)** Network visualization of keyword co-occurrence analysis generated by VOSviewer. Keywords with close associations are grouped into clusters, each denoted by a distinct color. **(B)** Overlay visualization of keyword co-occurrence analysis created by VOSviewer. Node color corresponds to the average appearing year (AAY) of each keyword. Purple or blue nodes indicate keywords that appeared relatively early in the field, while yellow-coded keywords highlight current research focuses. **(C)** Top 25 keywords with the strongest citation bursts identified by CiteSpace.

First, in the keyword co-occurrence network (**Figure 8A**), VOSviewer was employed to assign closely related keywords into clusters of the same color. After manually merging synonymous keywords and removing irrelevant ones, a total of 1,823 keywords were identified, representing the themes of the articles. Of these, 217 keywords appeared at least three times. The top five most frequently occurring keywords were: Immunotherapy, Immunohistochemistry, Microsatellite Instability, Tumor Immune Microenvironment, and Checkpoint Inhibitors. Additionally, VOSviewer automatically categorized all keywords into several main clusters. As shown in the figure, the keywords are divided into three major clusters: Cluster 1 (red nodes, Target Therapy/Checkpoint Inhibitors), Cluster 2 (green nodes, Tumor Immune Microenvironment/Immune Infiltration), and Cluster 3 (blue nodes, Estrogen/Progesterone Receptor). The profiles of the top 5 papers with the most citations in three clusters were shown in **Table 7**. Moreover, in the overlay visualization of keyword co-occurrence (**Figure 8B**), VOSviewer assigned different colors to keywords based on their average appearing year (AAY). Nodes marked in purple or blue represent keywords that emerged earlier, whereas those coded in yellow indicate the current research focuses. The overlay visualization (**Figure 8B**) reveals that current trending topics are primarily concentrated in Cluster 2.

**Table 7:** Top 5 papers from the first 3 clusters.

Cluster/Rank	Title	NC	Profile	RF
Cluster 1 Rank 1	Confirmation of ProMisE: a simple, genomics-based clinical classifier for endometrial cancer	567	This study confirms the feasibility and prognostic superiority of the ProMisE system, which categorizes endometrial carcinomas into four molecular subgroups, improving survival prediction, clinical management, and therapy guidance.	(51)
Cluster 1 Rank 2	Molecular classification of the portec-3 trial for high-risk endometrial cancer: impact on prognosis and benefit from adjuvant therapy	427	The PORTEC-3 trial demonstrated that molecular classification of high-risk endometrial cancer has strong prognostic value, with adjuvant chemoradiotherapy significantly improving recurrence-free survival in p53 abnormal tumors, highlighting the need for molecular-based risk stratification.	(53)
Cluster 1 Rank 3	Current recommendations and recent progress in endometrial cancer	392	Endometrial cancer, the most common gynecologic cancer in the U.S., faces treatment controversies, but advances include FDA-approved pembrolizumab for microsatellite-instable metastatic cases and ongoing trials to improve outcomes.	(55)
Cluster 1 Rank 4	Pembrolizumab in microsatellite instability high or mismatch repair deficient cancers: updated analysis from the phase II KEYNOTE-158 study	148	The KEYNOTE-158 study showed that pembrolizumab offers meaningful and durable benefits, with a 30.8% response rate and a 47.5-month median response duration, in heavily pretreated advanced MSI-H/dMMR non-colorectal cancers, with manageable safety.	(61)
Cluster 1 Rank 5	The epidemiology of endometrial and ovarian cancer	147	This review identifies shared risk factors for endometrial and ovarian cancers, highlighting estrogen excess, ovulatory cycles, immune mechanisms, and preventive measures like weight control and breastfeeding.	(62)
Cluster 2 Rank 1	Association of polymerase e-mutated and microsatellite-instable endometrial cancers with neoantigen load, number of tumor-infiltrating lymphocytes, and expression of PD-1 and PD-L1	480	POLE-mutated and MSI endometrial cancers are associated with high neo-antigen loads and abundant TILs, accompanied by overexpression of PD-1/PD-L1, making them ideal candidates for PD-1 targeted immunotherapy.	(52)
Cluster 2 Rank 2	Assessing tumor-infiltrating lymphocytes in solid tumors: a practical review for pathologists and proposal for a standardized method from the international immuno-oncology biomarkers working group: part 2: tils in melanoma, gastrointestinal tract carcinomas, non-small cell lung carcinoma and mesothelioma, endometrial and ovarian carcinomas, squamous cell carcinoma of the head and neck, genitourinary carcinomas, and primary brain tumors	405	This review proposes a standardized methodology for assessing TILs and discusses their prognostic and predictive value across various solid tumors.	(54)
Cluster 2 Rank 3	Safety and antitumor activity of pembrolizumab in advanced programmed death ligand 1-positive endometrial cancer: results from the keynote-028 study	352	The KEYNOTE-028 study showed that pembrolizumab is safe and effective for heavily pretreated PD-L1-positive advanced endometrial cancer, with durable antitumor activity and manageable side effects.	(57)
Cluster 2 Rank 4	Canonical and non-canonical WNT signaling in cancer stem cells and their niches: Cellular heterogeneity, omics reprogramming, targeted therapy and tumor plasticity	320	This review discusses WNT signaling’s role in cancer stem cell survival, metastasis, and resistance, highlighting targeted therapies in clinical trials for WNT-driven cancers.	(63)
Cluster 2 Rank 5	Landscape of phosphatidylinositol-3-kinase pathway alterations across 19 784 diverse solid tumors	188	This study identifies common PI3K pathway aberrations in various cancers and highlights co-occurring hormone receptor and HER2 alterations for targeted therapy opportunities.	(64)
Cluster 3 Rank 1	Proteogenomic characterization of endometrial carcinoma	271	This study provides a comprehensive proteogenomic characterization of endometrial carcinomas, identifying novel molecular associations and potential therapeutic targets, including immune landscape insights.	(58)
Cluster 3 Rank 2	Molecular approaches for classifying endometrial carcinoma	139	This review highlights the integration of molecular techniques with histological classification to improve the prognosis and treatment of endometrial carcinoma, including immunotherapy guidance.	(65)
Cluster 3 Rank 3	Estrogen receptor β: the guardian of the endometrium	105	This review highlights protective role of Erβ in the human endometrium and its potential involvement in regulating cell fate and endometrial diseases.	(66)
Cluster 3 Rank 4	Molecular pathology of Lynch syndrome	99	Lynch syndrome is a common genetic predisposition to colorectal, endometrial, and other cancers, linked to DNA mismatch repair gene mutations.	(67)
Cluster 3 Rank 5	Polymerase ϵ (POLE) ultra-mutation in uterine tumors correlates with T lymphocyte infiltration and increased resistance to platinum-based chemotherapy *in vitro*	51	POLE-mutated endometrial carcinomas have improved prognosis due to high TIL infiltration and immunogenicity, not chemotherapy sensitivity.	(68)

Ranking: according to the number of total publications. NC, total number of citations; RF, references.

Using CiteSpace, the top 25 burst keywords were analyzed (**Figure 8C**). Notable among these are keywords whose bursts have persisted to the present, including “Tumor Microenvironment” (burst strength 3.38), “Uterine Corpus Endometrial Carcinoma” (burst strength 3.75), “Immune Infiltration” (burst strength 2.93), “Molecular Classification” (burst strength 2.88), and “Carboplatin” (burst strength 1.98).

## 4 Discussion

The immunotherapy of tumors has evolved from theoretical hypotheses to continuous refinement and breakthroughs in clinical applications. As early as 1909, Paul Ehrlich proposed the hypothesis that the immune system could recognize and eliminate tumor cells, laying the foundation for tumor immunology research (69). At the end of the 19th century, William B. Coley attempted to stimulate the immune system using bacterial toxins to treat cancer, marking the prototype of immunotherapy (70). In the 1960s, Burnet and Thomas introduced the “immune surveillance theory,” uncovering the mechanism of tumor immune evasion (71). In the 1970s, interleukin-2 (IL-2) and interferons were employed in cancer treatment, driving the development of non-specific immunotherapy (72). In 1997, the first monoclonal antibody for cancer treatment, rituximab, was approved, heralding a new era of targeted immunotherapy (73). Subsequently, drugs such as trastuzumab were widely applied to breast cancer and lymphoma (74). In-depth research on tumor antigens has led to the development of antigen-based vaccines. Key identification methods include gene expression analysis, proteomics, and immunohistochemistry for cancer-testis (CT) antigens, and whole-exome sequencing, bioinformatics, and immunological validation for neoantigens (75, 76). Researchers have used CT antigens as targets for tumor vaccines and developed personalized vaccines by identifying patient-specific neoantigens (77). These strategies have diversified tumor immunotherapy approaches, advancing the development of vaccines for malignant tumors like melanoma (77, 78). In 2011, ipilimumab, the first immune checkpoint inhibitor, was approved, followed by the emergence of PD-1/PD-L1 inhibitors, which significantly prolonged survival in various cancer patients and ushered in a new era of tumor immunotherapy (79). In 2017, CAR-T cell therapy was approved, bringing revolutionary progress in treating hematologic malignancies (38). In recent years, personalized vaccines based on neoantigens and strategies to regulate the tumor microenvironment have become research hotspots, driving immunotherapy toward precision medicine (80, 81).

This study conducted the first bibliometric analysis of EC immunotherapy-related literature published between 2012 and August 2024. Relevant papers were retrieved from the WoSCC database and analyzed using advanced software tools to construct and visualize bibliometric networks, deeply exploring research trends and development patterns in this field. The comprehensive application of these methods provides critical support for systematically understanding the research landscape and overall structure of this domain.

In general, the annual number of publications and citations are the most direct and effective indicators of scholars’ research focus in a particular field (82). According to our model analysis, in 2023, the number of publications in this field reached 152, while citations surpassed 3,623, both setting historical records for the field. This growth not only indicates that research in this area is receiving increasing attention but also reflects the growing interest and investment from the academic community in this topic. So far, the total number of citations for publications in this field has accumulated to 18,344, demonstrating the significance of these research results within the academic world. It is expected that with the emergence of more innovative findings, research in this field will reach new heights in 2024 and continue to grow. Further analysis reveals that over the past 13 years, both the annual publication volume and total citations have shown a significant upward trend, which not only affirms the progress of research in this field but also indicates the widespread attention and interest it has generated within both the academic and research communities (83).

Tracking the contributions of countries, institutions, and research teams provides valuable insights into the research trends in this field. Globally, China and the United States rank first and second in terms of publication volume, with 353 and 202 papers, respectively. This high publication output reflects a greater societal demand for related research in these countries compared to others. However, the United States stands out in terms of academic influence and innovation, acting as a leading pioneer in this field. In contrast, China should focus on enhancing its international accessibility through universities and research institutions to optimize research strategies and expand its influence. As globalization progresses, international collaboration is becoming increasingly important. The high academic influence of the United States is partly attributed to its effective cooperation with other countries, while collaboration between certain nations remains insufficient. Strengthening international partnerships could be an effective way to deepen research and applications in this field. Collaboration facilitates knowledge exchange, innovative ideas, and resource integration, ultimately advancing discipline.

Among the institutions with the highest publication volumes, the University of Texas Medical Anderson Cancer Center and Nanjing Medical University tie for first place, each with 26 papers. They are followed by Zhejiang University (22 papers), Fudan University (21 papers), and Leiden University (19 papers) (**Figure 2B**, **Table 1**). In terms of average citations per paper, Nanjing Medical University achieves 13.00 citations, while Leiden University and the University of Texas Medical Anderson Cancer Center lead with 53.84 and 39.69 citations, respectively. These findings align with national-level research outcomes. Thus, national-level studies must not only enhance research depth but also improve the international visibility of their publications to boost their impact. Additionally, results show that leading institutions, both domestic and international, remain the driving forces in academic research. Regarding funding agencies, the United States and Japan each account for 30% of the top 10 active funders, while China contributes 20% (**Figure 3A** and **Table 2**). However, the extent of international collaboration between institutions remains insufficient, with most partnerships occurring within national boundaries. This highlights the need for stronger international collaboration among institutions.

Through publication analysis, *Gynecologic Oncology* is identified as the most influential journal in the field, while *Cancers*, *Frontiers in Oncology*, *Frontiers in Immunology*, and *Frontiers in Genetics* are recognized as promising journals in the area of EC immunotherapy (**Figure 3B** and **Table 4**). Scholars from Leiden University, particularly the team of Bosse and Tjalling, lead in publications within the field with 18 papers, achieving the highest total citations (909) and total link strength (99). Notably, five of the top 10 authors are from this team, underscoring their sustained impact in this field (**Table 5**). Additionally, Gynecologic Oncology stands out with the highest total link strength (90,114) and citation count (2,456), further establishing its leading role in the field and providing critical references for future research and publication.

Highly cited papers reveal key research hotspots in the field. The paper by Talhouk et al. ranks first, with an average of 70.88 citations per year (**Table 6**). This study introduced the ProMisE molecular classification system based on The Cancer Genome Atlas (TCGA) and validated its feasibility and prognostic capability in a large EC cohort, providing a novel method for molecular classification and prognosis prediction in EC patients (51). The team led by Howitt focused on the application of immune checkpoint inhibitors in EC. Using TCGA data, they predicted neo-antigen loads and evaluated tumor-infiltrating lymphocytes (TILs) and PD-1/PD-L1 expression in 63 EC patients. Their findings demonstrated that EC tumors with POLE mutations and MSI are associated with high neoantigen loads and TILs, along with PD-1/PD-L1 overexpression, making them promising candidates for PD-1-targeted immunotherapy (52). Leon-Castillo et al. conducted studies on high-risk EC patients, comparing the efficacy of combined chemoradiotherapy (CTRT) versus radiotherapy (RT) alone. Based on molecular classification, they evaluated the impact of chemotherapy on different subgroups and found that for p53abn tumors, CTRT significantly improved RFS, while POLEmut patients exhibited excellent RFS in both treatment regimens. They suggested future risk stratification and clinical trials should incorporate molecular classification to tailor treatments for specific subgroups (53). Overall, these highly cited research directions provide comprehensive guidance for the discipline’s frontiers and hotspots, holding significant reference value.

Research focal points can be effectively identified through the analysis of keywords and references, offering valuable insights into cutting-edge research directions and emerging trends. As illustrated in **Figure 6A**, references are grouped into nine distinct clusters. While earlier research predominantly explored areas such as “Micrometastases,” “Estrogen Receptor Alpha,” and “Endometrial Hyperplasia,” recent studies have shifted focus toward emerging topics, including “Ferroptosis-Related lncRNA,” “Tumor Mutation Burden,” and “PD-L1 Checkpoint Inhibition.” Subsequent biplot analysis (**Figure 6B**) reveals that articles in domains like Molecular Biology, Genetics, and Health, Nursing & Medicine are primarily cited by journals in Molecular Biology, Immunology, and Clinical Medicine. The growing citation links from medical and clinical sources highlight the increasing importance of interdisciplinary collaboration in advancing the transition from fundamental research to practical applications.

The analysis of reference citation bursts identifies articles that experienced notable surges in citations during specific periods, signaling rapid recognition and dissemination within the research community. The top 25 citation bursts (**Figure 7B**) indicate that the earliest burst occurred in 2014, while the most recent appeared in 2023. Moreover, keyword analysis offers further insights by identifying emerging topics and predicting future research trajectories. In this study, 141 critical keywords were identified and categorized into three main clusters. As shown in **Figure 8A**, these clusters include Cluster 1 (red nodes), focusing on Targeted Therapy and Checkpoint Inhibitors; Cluster 2 (green nodes), emphasizing the Tumor Immune Microenvironment and Immune Infiltration; and Cluster 3 (blue nodes), centered on Estrogen and Progesterone Receptors. Additional analysis (**Figure 8B**) highlights current research hotspots, such as “Checkpoint Inhibitors,” “m6A Modifications,” “Machine Learning,” “Cuproptosis,” and “Pyroptosis.”

To further examine emerging trends, we analyzed the top 25 keywords (**Figure 8C**), which primarily include “Tumor Microenvironment,” “Immune Infiltration,” and “Immune Checkpoint Inhibitors.” These keywords underscore their persistent relevance and focused attention within outbreak-related research, aligning closely with the findings of the citation burst analysis. The most highly cited papers in these three clusters are detailed in **Table 7**, offering a comprehensive overview and pioneering insights into this field. By examining the content and perspectives of these papers, researchers can gain a deeper understanding of current trends and valuable guidance for future investigations.

In recent years, immunotherapy research for EC has made significant progress, focusing on the tumor immune microenvironment (TME), molecular modifications, regulatory mechanisms, and the integration of emerging technologies. These advancements have introduced novel perspectives and strategies for EC treatment, particularly in unraveling tumor immune evasion mechanisms and enhancing immunotherapy efficacy (84).

The TME plays a critical role in the initiation, progression, and immunotherapeutic response of EC. Comprising tumor cells, immune cells, stromal cells, vasculature, and cytokine networks, the TME’s immune cell infiltration notably influences tumor immune evasion and therapy outcomes. Studies highlight the prominent roles of tumor-associated macrophages (TAMs), regulatory T cells (Tregs), and dysfunctional effector T cells in the EC TME (85). These cells drive immune evasion through mechanisms such as the secretion of immunosuppressive cytokines (e.g., IL-10 and TGF-β) and the upregulation of immune checkpoint molecules (e.g., PD-L1). Additionally, TAM polarization correlates strongly with prognosis: M2 macrophages are linked to immunosuppression and poor outcomes, while M1 macrophages exhibit antitumor properties. Modulating immune cell proportions and activity in the TME, through immunomodulators or combined chemo-radiotherapy, has demonstrated potential to improve immunotherapeutic outcomes, laying the groundwork for TME-targeted immunotherapy strategies (28).

Immune checkpoint inhibitors (ICIs) have revolutionized cancer immunotherapy, showing notable success in EC treatment. Anti-PD-1/PD-L1 and anti-CTLA-4 monoclonal antibodies have been particularly effective for patients with MSI-H or dMMR EC (4). However, their efficacy in microsatellite-stable (MSS) EC remains limited due to low T-cell infiltration and elevated expression of immunosuppressive molecules in the TME. To address these challenges, researchers are exploring combination therapies. For example, combining anti-PD-1/PD-L1 antibodies with anti-angiogenic agents like bevacizumab can enhance T-cell infiltration by normalizing the tumor vasculature. Additionally, combining ICIs with chemotherapy, radiotherapy, or targeted therapies is under investigation, aiming to induce immunogenic cell death or bolster antitumor immune responses (35). These approaches offer promising pathways to enhance ICI efficacy in MSS patients.

Chimeric antigen receptor T-cell (CAR-T) therapy is in its nascent stages for EC treatment. Preclinical studies targeting tumor-associated antigens such as MUC1 and HER2 have demonstrated promising anti-improvement activity by adoptive T cell therapy (86, 87). However, clinical application faces challenges such as tumor heterogeneity and immunosuppressive TME (88). To overcome these barriers, researchers are advancing next-generation CAR-T technologies, including dual-specific CAR-T cells targeting multiple antigens and gene-edited CAR-T cells optimized for survival in suppressive environments (39). Combining CAR-T therapy with ICIs or other immunomodulators is also being explored as a potential solution. For example, ICIs block inhibitory signals such as PD-1 and CTLA-4 to prevent T cell exhaustion and enhance anti-tumor effects (35). They reshape the tumor microenvironment, reduce immune suppression, and promote CAR-T cell infiltration and function. Additionally, precise immune regulation helps minimize cytokine release syndrome and other adverse effects, improving safety (38, 79). The synergy of these mechanisms is expected to enhance the efficacy and safety of CAR-T therapy in EC treatment (3, 24).

Epigenetic modifications are increasingly recognized as key players in immune regulation in EC, with methylation and phosphorylation mechanisms being particularly significant. Aberrant DNA methylation can promote immune evasion by silencing immune-related genes, while hyperactive phosphorylation pathways may enhance TME immunosuppression (89). Other epigenetic mechanisms, such as protein acetylation and histone modifications, also warrant further investigation for their roles in tumor immunity (90). Additionally, emerging forms of programmed cell death, such as pyroptosis and cuproptosis, provide new opportunities for inducing tumor immune responses. Pyroptosis, a pro-inflammatory cell death pathway, activates antitumor immunity by releasing immunogenic molecules, while cuproptosis disrupts tumor cell growth through copper ion metabolism regulation (91, 92). Therapeutic strategies leveraging these mechanisms hold potential for significantly enhancing immunotherapy efficacy.

Nanotechnology has also gained attention in immunotherapy applications. Nanoparticles, as drug delivery vehicles, offer advantages in efficient delivery and targeting. For instance, nanoparticles delivering antigens or immunoadjuvants can amplify antitumor immune responses, while CRISPR/Cas9-based nanotechnology enables precise gene editing (93, 94). Nanotechnology-based immunotherapy can also be combined with other treatments, such as photodynamic therapy (PDT) or photothermal therapy (PTT), for improved outcomes (95, 96). Meanwhile, machine learning is emerging as a powerful tool for personalized EC treatment. By integrating multi-omics data (e.g., genomic, transcriptomic, and epigenomic data), machine learning models can predict patient responses to immunotherapy and guide precise drug selection. This combination of big data analytics and artificial intelligence offers a promising avenue for developing more effective, personalized treatment strategies (97, 98).

Overall, EC immunotherapy is advancing toward a multidimensional and integrative paradigm. From dynamic TME regulation and epigenetic exploration to CAR-T therapy optimization and nanotechnology innovation, the field is thriving. However, challenges remain in clinical translation, including treatment safety, efficacy, and cost-effectiveness. Future research should prioritize: (1) Mechanistic Insights: Deepening understanding of immune-tumor interactions within the TME, particularly the immune evasion mechanisms in MSS EC with low immune infiltration. (2) Optimized Combination Therapies: Identifying optimal regimens for ICIs combined with other modalities and expanding clinical trials for low-immune-response patients. (3) Technological Integration: Leveraging machine learning, nanotechnology, and epigenetics to advance precision immunotherapy. (4) Emerging Mechanisms in Practice: Translating novel mechanisms, such as pyroptosis and cuproptosis, into safe and effective clinical strategies.

In conclusion, this bibliometric analysis provides a clear framework to guide future efforts in the pathophysiology, molecular biology, and clinical research of EC immunotherapy. By fostering collaboration and integration across institutions, research teams, and funding bodies, while keeping pace with emerging priorities, EC immunotherapy can advance from foundational research to clinical application, ultimately benefiting a broader patient population.

## 5 Limitations

This study has several limitations that require attention. Firstly, the data source was limited to WoSCC, which, although extensive, may not encompass all relevant publications. Secondly, the quality of the included literature varied considerably, potentially introducing biases and affecting the overall reliability of the analysis results. Moreover, it should be recognized that all bibliometric tools have inherent limitations. During the process of extracting terms from titles, abstracts, and keywords, the results of cluster analysis may fluctuate significantly, and it cannot be fully ensured that terms with similar meanings are accurately consolidated. Although this study enhanced its objectivity and reliability through preregistration and blind design, it is still important to acknowledge that the validity of the research model depends on the selected screening criteria and the quality of the retrieved records. Therefore, future studies should consider integrating multiple databases and standardized tools to gain a more comprehensive and in-depth understanding of global research on EC immunotherapy, thereby forming a more complete and systematic perspective.

## 6 Conclusion

As far as we are aware, this study represents a novel comprehensive bibliometric analysis of immunotherapy for EC, offering insights into the current research progress and global trends in this field. The findings reveal that this area is rapidly evolving and is expected to continue expanding in the future. China and the United States are the primary driving forces, holding central positions in global research on this topic. To accelerate progress, future researchers should prioritize fostering greater collaboration across countries and regions. Institutions such as the Texas Medical Anderson Cancer Center and Nanjing Medical University, along with the journal *Gynecologic Oncology*, have demonstrated exceptional productivity and influence. Research teams led by Bosse, Tjalling, and Creutzberg, Carien L have been pivotal in advancing the field.

Currently, key research hotspots include the “Tumor Immune Microenvironment, “ “Immune Checkpoint Inhibitors,” and “Targeted Therapy.” Looking ahead, promising research directions may focus on further exploring the underlying mechanisms, optimizing combination therapy strategies, and integrating cross-disciplinary technologies to drive the transition of EC immunotherapy strategies from basic research to clinical applications.

In conclusion, this bibliometric analysis provides a comprehensive overview of research in EC immunotherapy, offering valuable insights for researchers and decision-makers while playing a crucial role in guiding the translation of foundational research into practical applications in this field.

## Data Availability

The raw data supporting the conclusions of this article will be made available by the authors, without undue reservation.
